# Pathogen and Host Response Dynamics in a Mouse Model of *Borrelia hermsii* Relapsing Fever

**DOI:** 10.3390/vetsci3030019

**Published:** 2016-08-30

**Authors:** Christopher D. Crowder, Arash Ghalyanchi Langeroudi, Azadeh Shojaee Estabragh, Eric R. G. Lewis, Renee A. Marcsisin, Alan G. Barbour

**Affiliations:** Departments of Microbiology & Molecular Genetics and Medicine, University of California Irvine, Irvine, CA 92697, USA; ccrowder@gmail.com (C.D.C.); arashghalyanchi@gmail.com (A.G.L.); ashojaiestabragh@gmail.com (A.S.E.); erlewis@utmb.edu (E.R.G.L.); reneemarcsisin@gmail.com (R.A.M.)

**Keywords:** tick-borne disease, zoonosis, spirochete, antigenic variation, *Ornithodoros*, *Mus musculus*

## Abstract

Most *Borrelia* species that cause tick-borne relapsing fever utilize rodents as their natural reservoirs, and for decades laboratory-bred rodents have served as informative experimental models for the disease. However, while there has much progress in understanding the pathogenetic mechanisms, including antigenic variation, of the pathogen, the host side of the equation has been neglected. Using different approaches, we studied, in immunocompetent inbred mice, the dynamics of infection with and host responses to North American relapsing fever agent *B. hermsii*. The spirochete’s generation time in blood of infected mice was between 4–5 h and, after a delay, was matched in rate by the increase of specific agglutinating antibodies in response to the infection. After initiating serotype cells were cleared by antibodies, the surviving spirochetes were a different serotype and, as a population, grew more slowly. The retardation was attributable to the host response and not an inherently slower growth rate. The innate responses at infection peak and immediate aftermath were characterized by elevations of both pro-inflammatory and anti-inflammatory cytokines and chemokines. Immunodeficient mice had higher spirochete burdens and severe anemia, which was accounted for by aggregation of erythrocytes by spirochetes and their partially reversible sequestration in greatly enlarged spleens and elsewhere.

## 1. Introduction

### 1.1. Early Animal Experiments

Relapsing fever was one of the first diseases for which the cause, the etiology, was identified. In 1868 Otto Obermeier, a junior-level physician at a Berlin hospital, observed thin “threads” with “corkscrew”-like shapes and undulating and “locomotive” movements in the blood of several patients with relapsing fever during an epidemic. The motile organisms were among the blood “corpuscles” at the times of fever in the patients but were consistently undetectable during remission. This observation and his subsequent studies were published by Obermeier in 1873 in a German medical journal [1], and then reported by Fitz in digest form in English later that year [2]. Obermeier also carried out the first experimental animal studies of relapsing fever. He injected the blood of patients into dogs, guinea pigs, and rabbits, but was not able to reproduce the infection in any of these species. This is likely because his patients had epidemic or louse-borne relapsing fever and were thus infected with *Borrelia recurrentis*, whose host range in nature is effectively restricted to humans [3]. Motschutkoffsky could experimentally reproduce the illness and Obermeier’s microbiological findings but only by injecting healthy individuals and himself with blood from patients with relapsing fever [4].

If Obermier’s relapsing fever cases were tick instead of louse in origin, his attempts to infect adult non-primate animals with a *Borrelia* species probably would have succeeded. However, it would be another three decades before “tick fever” in central Africa was attributed to a similar but distinct organism from the “*Spirillum obermeieri*” (*B. recurrentis*) which was causing relapsing fever in its epidemic form in Europe and Asia at the time. The disease in sub-Saharan Africa, as it was described in a tropical disease textbook of the time [5], featured recurrent febrile episodes, and it was associated with the bites of the soft tick *Ornithodoros moubata* [6]. Ross and Milne [7] and Dutton and Todd [8] independently demonstrated that “tick fever” was caused by “spirilla” in the blood. Dutton and Todd were able to “uniformly” infect *Cercopithecus* sp. monkeys but inconsistently guinea pigs. This host range profile for the “spirillum” is consistent with adaptation of what was likely *Borrelia duttonii* for exploitation of humans as a major if not sole reservoir host [9]. Contemporary studies by Robert Koch demonstrated transovarial transmission of the organisms in the tick vector [10].

In the year following publications of these seminal studies in Africa, Robert Carlisle in New York City reported a case of relapsing fever and the isolation of spirochetes, which had been observed in the patient’s blood smear, by inoculation of a blood sample into a Rhesus macaque [11]. From Carlisle’s history of the case, the patient likely acquired the infection in Texas in North America, not in Africa, Europe, or Asia. Breinl reported that this North American agent of “tick fever” was serologically distinguishable from the African variety [12].

Norris et al. in turn used this isolate and confirmed the susceptibility of macaques and replicated the relapsing course under experimental conditions [13]. Norris et al. also carried out experiments with large numbers of white rats, which routinely had “spirillosis” of the blood within one to five days of subcutaneous inoculation of infected macaque blood. A characteristic feature of the infected rats was splenomegaly. The authors also successfully infected “white mice” and some rabbits but not guinea pigs. In both macaques and rats, previous infection conferred immunity to challenge with the same isolate. 

Novy and Knapp in Michigan also obtained Carlisle’s isolate and carried out an extensive and well-documented series of experiments with animals [14]. Rats and mice were highly susceptible to infection, but rabbits and guinea pigs were relatively or absolutely resistant, a host range profile compatible with *Borrelia turicatae* [15,16]. The identity of “Spirochaeta novyi” with *B. turicatae* was confirmed by Brumpt [17].

Once these researchers in the early 1900s demonstrated experimental models of relapsing fever in standard laboratory animals, such as mice and rats, as well as primates, hundreds of published studies on this subject in several languages followed over the succeeding decades. Many of these were reviewed in one or more of References [18,19,20,21,22,23,24,25,26]. Here, we focus on one of the *Borrelia* species that cause tick-borne relapsing fever: *B. hermsii*. One justification for this emphasis is the arguably greater cumulative knowledge about this species in the laboratory up to this point. 

### 1.2. The North American Species B. hermsii

In the 1930s *B. hermsii* and the tick *Ornothodoros hermsi* were identified as, respectively, the cause and arthropod vector of cases of relapsing fever in California [27,28]. *B. hermsii* is also found in mountains and foothills elsewhere in western North America, from British Columbia in the north, through the Great Basin region and the Rocky Mountains, and south into Arizona and New Mexico [29]. Reports of *B. hermsii* relapsing fever have included cases among residents and visitors of the Sierra Nevada mountains of California since 1922 [30] and of the Grand Canyon National Park in Arizona [31]. The usual reservoir hosts for *B. hermsii* are chipmunks and squirrels [32,33], but can also include *Peromyscus* spp., such as the deer mouse [34].

On the basis of whole-genome as well as shorter sequences, *B. hermsii*, together with *B. turicatae*, *B. parkeri*, and *B. coriaceae*, constitute a North American or “New World” clade [35]. Another set of species, including *B. duttonii*, *B. crocidurae*, *B. hispanica*, and *B. persica*, in Africa and Eurasia coherently constitute an “Old World” taxonomic clade [36]. The sole louse-borne relapsing fever agent, *B. recurrentis*, has a reduced genome and is a close derivative of *B. duttonii* [37]. There are other species (e.g., *B. miyamotoi*) in the combined relapsing fever group in the genus *Borrelia* that are carried hard ticks, such as *Ixodes scapularis*, instead of soft ticks, and are found in North America as well as Eurasia [35], but these are not further considered here. *B. anserina* is another species in the relapsing fever group and is transmitted by soft ticks of the genus *Argas*, but its host range is largely restricted to birds, and recurrences of the bacteremia have not been observed [38]. 

Following *B. hermsii*’s identification in California, this organism and other relapsing fever agents were studied by Gordon Davis and Willy Burgdorfer at the National Institutes of Health’s Rocky Mountain Laboratory (RML) (e.g., [39,40]), by Dorthy Beck in California [41], and later by Coffey and Eveland in California [42,43]. Richard Kelly’s breakthrough in cultivating a relapsing fever agent in the laboratory was achieved with *B. hermsii* [44]. Herbert Stoenner at the RML improved Kelly’s medium [45] and used a strain, now named HS1, which was originally isolated in mice by Willy Burgdorfer from ticks collected at the site of an outbreak of relapsing fever in eastern Washington in 1968 [46]. Stoenner and Barbour used clonal populations of strain HS1 and variant-specific antibodies in their studies of antigenic variation during relapsing fever [47,48]. Schwan and his colleagues in their in-depth studies of the biology of *B. hermsii* at the RML used isolate HS1 through 1998 [49], but since 2000 they have primarily used isolate DAH, which has a different origin than HS1 [50]. However, the DAH and HS1 isolates are near-identical in sequence and essentially are the same strain [51,52]. In one study DAH and HS1 isolates reached the same peak densities in the blood of infected mice [53].

### 1.3. Overview

Two major aims of the paper are, first, to report on a series of experiments we have carried out that further define the biology of *B. hermsii* infection of *Mus musculus* in the laboratory, and, second, to put these findings in the context of previous work, with an emphasis, as for this introduction, on early investigations that have stood up over time. Inclusion of all pertinent studies of relapsing fever in animal models—a century-long, global endeavor—was beyond the scope of our more limited ambition. Many exemplary articles of our predecessors and contemporaries in the field could not be included. Mechanisms of disease are not neglected, but attention is focused primarily on the dynamics of growth and responses and the variances for the dynamics. Another objective is the provision of empirical data for those who would build deterministic or agent-based models of relapsing fever that incorporate both pathogen and host parameters. Although most of the space is devoted to experiments in laboratory animals and most other references are to human infection, there is increasing recognition of the occurrence of tick-borne relapsing fever in dogs and cats [54,55,56,57]. The studies of experimental animal models of relapsing fever likely provide insights for research on and clinical management of relapsing fever in domestic and companion animals.

## 2. Materials and Methods

### 2.1. Animals

The vertebrate animals protocol was approved by the Institutional Animal Care and Utilization Committee of the University of California Irvine (Approval code: 2080-1999). Inbred *Mus musculus* mice were strain BALB/c (Charles Rivers Laboratories) and two strains congenic with BALB/c with the severe combined immunodeficiency phenotype (SCID) and mutation (*scid*): CBySmn.CB17*-Prkdc^scid^*/J from Jackson Laboratory (BALB/c *scid*) and CB17/lcr-*Prkdc^scid^*/IcrlcoCrl from Charles River Laboratories (C.B-17 *scid*). Other mice were Nu/J nude mice from Jackson Laboratory. Immunodeficient mice were housed in isolator cages under ABSL2 containment in an ALAAC-approved facility, provided with autoclaved bedding and food (Harlan Teklad Global Soy Protein-Free Rodent Diet), were kept on a 12 h light-dark cycle, and received autoclaved distilled water ad libitum. During experiments mice were examined and weighed daily. Blood was collected from either the tail (10–25 μL) or the saphenous vein (50 μL) in lithium-heparin coated Microvette CB300 collection tubes (Sarstedt). Terminal exsanguination and euthanasia under isofluorane or CO_2_ anesthesia was performed by cardiac puncture and collection of the blood into either a heparinized syringe or heparinized tubes (Becton-Dickinson Microcontainer #365965).

### 2.2. Bacterial Strains and Culture Conditions

The Browne Mountain isolate of the type strain HS1 of *B. hermsii* (ATCC 35209; BioSample SAMN04481062) was used [46,52]. Original frozen stocks of mouse plasma with either 25% glycerol or 10% dimethyl sulfoxide were ≥98% pure in serotype identity by immunoflourescence assay with serotype-specific antisera [47]. For the present study, serotype 7 from these stocks was cloned again by limiting dilution in C.B-17 *scid* mice. The serotype of a relapse population was identified by PCR amplification and sequence of the expression site for the variable major proteins, as described [58,59]. Two other serotypes of strain HS1 used in the study were serotype 19 and serotype 33. The second *B. hermsii* strain was CC1 serotype 1 (BioSample SAMN03408291) [59,60], which was first passed into C.B-17 *scid* mice from frozen stocks before inoculation of the freshly-obtained plasma into other sets for mice for experiments. *B. hermsii* cells were cultivated in BSK II medium with 12% rabbit serum at 34 °C unless otherwise stated [61]. Bacteria were harvested from cultures and then washed as described [59]. Spirochetes in plasma or culture medium were counted in duplicate by phase microscopy on an Olympus BX40 microscope using a Petroff-Hausser counting chamber with a depth of 0.02 mm and 400× magnification. A volume of 4.5 μL was placed into the chamber and spirochetes in its 400 squares (1 mm^2^ total area) were counted.

### 2.3. Mouse Infections

Mice were inoculated intraperitoneally with 1–10 *B. hermsii* cells in 100 μL of phosphate-buffered saline, pH 7.4 (PBS), with 5 mM MgCl_2_ (PBS-Mg) by intraperitoneal injection, unless otherwise noted. The innoculum cell count was determined either by limiting dilution for frozen stock of known viability or microscopic quantitation of cultures as described above. A rotation of one or two members of each group of inoculated mice was monitored daily for the presence and density of spirochetes by phase-contrast microscopy of a wet mount of blood obtained from the tail vein. Infected plasma was obtained from blood anti-coagulated with heparin or sodium citrate by centrifugation at 100× *g* for 3 min. Cell-free plasma was obtained by centrifugation of the infected plasma at 9500× *g* for 5 min for the aggregation experiment and at 16,000× *g* for 10 min for antibody experiments. At the time terminal anesthesia, the mice were weighed, and whole spleens were dissected and weighed. Spleens of some mice were fixed in 10% buffered formalin for histopathology processing at University of California Davis’ Comparative Pathology Laboratory (Davis, CA, USA). Mice that were treated with an antibiotic received ceftriaxone (Sigma-Aldrich) at a dose of 25 µg per gm of body weight and administered subcutaneously every 12 h for 3 d, as described [62]. The hematocrit, percentage of packed red blood cells, was determined with heparinized microhematocrit capillary tubes (Fisher Scientific) with Critoseal caps (Oxford Labware, St. Louis, MO, USA) and centrifugation for 5 min on a ZIPocrit microhematocrit centrifuge (LW Scientific, Lawrenceville, GA, USA).

### 2.4. Antibody Agglutination Assays

The macro-agglutination and micro-agglutination assays were performed in 96-well, round-bottom, polystyrene 96-well microtiter plates. To each of the wells, which contained 25 µL of PBS-Mg with 5% bovine serum albumin (BSA) and 10^7^ in vitro—cultivated or plasma-borne bacteria, was added an equal volume of mouse plasma serially two-fold diluted in the same buffer. Reactions were shaken at 200 rpm and incubated at 37 °C for 2 h. The highest dilution in which there was cell pellet surrounded by clear liquid rather than a homogeneous haze was recorded as the titer of the assay. For the micro-agglutination assay 12.5 µL of plasma serially-diluted in BSK II medium were added to 12.5 μL of a suspension of bacteria at a concentration of 5 × 10^7^ per mL of medium. The suspension was incubated at 37 °C for 2 h on a shaker at 200 rpm. A 5 μL volume was examined under a cover slip by phase microscopy at 400× magnification. Agglutination was scored positive if >50% of the spirochetes were in clumps of ≥5 cells. A positive control for the micro-agglutination was a mouse monoclonal antibody H7-7 to serotype 7 with documented agglutinating capacity [63].

### 2.5. Growth Inhibition Assay

The growth inhibition assay for *B. hermsii* HS1 serotype 33 was performed in 200 μL reaction volumes in a 96-well microtiter plate as described [63]. A two-fold dilution series was made for both *B. hermsii* serotype 33 cells in BSKII media and monoclonal antibody H4825 diluted in BSK II media, IgG2a antibody specific for the Vtp protein of serotype 33 [64]. Each reaction well consisted of 195 μL of *B. hermsii* cell dilution and 5 μL of antibody dilution in a checkerboard fashion. Guinea pig complement (Diamedix) at two units per mL was added to some wells. The plate was then sealed and incubated at 34 °C for two weeks. The plate was monitored daily for growth of *B. hermsii* cells as indicated by a change in media color from pink to yellow. Wells were considered positive for growth inhibition if the media remained pink.

### 2.6. Indirect Immunofluorescence Assay (IFA)

Methanol-fixed thin smears of blood from mice infected with *B. hermsii* were incubated with mouse plasma diluted 1:10 in PBS at 37 °C for 1 h. The slides were washed with PBS for twice for 10 min each, and then incubated with fluorescein-labeled goat anti-mouse IgM antibody (Kierkegaard & Perry Laboratory, Gaithersburg, MD, USA) at 1:1000 dilution at 37 °C for 1 h. After washing again with PBS, buffered-glycerol and a cover slip were applied, and the slides were examined by UV microscopy.

### 2.7. Enzyme-Linked Immunosorbent (ELISA) Assay for IgM Antibodies

To wells of a 96-well flat-bottom polystyrene microtiter plate were added 10^8^ washed bacteria suspended in 50 μL of 50 mM sodium carbonate, pH 9.6 buffer. Plate were centrifuged at 700× *g* for 30 min, buffer was removed by aspiration, wells were washed with 50 μL of PBS-Mg, and plates were centrifuged again at 700× *g* for 30 min. Wells were blocked with 200 μL blocking 50 mM Tris, pH 8.0-140 mM NaCl-1% BSA for 30 min. This solution was removed and the wells were washed three times with 50 mM Tris-140 mM NaCl, pH 8.0-0.05% Tween 20 (wash solution). Serial two-fold dilutions of the mouse plasma were made in the wash solution and added in 100 µL volumes to the wells of the plates, which were then incubated at 22 °C for 1 h. The wells were aspirated of their contents and then washed five times with the wash solution. Volumes of 100 μL of horse radish peroxidase-conjugated goat antibody for murine IgM (Bethyl Laboratories) at a 1:100,000 dilution in wash solution were then added to wells and incubation at 22 °C for 1 h was carried out. After the wells were aspirated, they were washed five times with wash solution. The reaction was developed with 3,3’, 5,5’-tetramethylbenzidine (TMB) of the ELISA Starter Accessory Kit (Bethyl Labs), and optical densities of the colorimetric reactions were read at 450 nm. The cutoff value for a positive titer was defined as >3 standard deviations above the mean of 16 plasma samples from uninfected mice.

### 2.8. Assays for Cytokines, Chemokines, and Other Serum Components

Samples of freshly-obtained heparinized mouse blood were centrifuged at 13× *g* for 10 min. The plasma was then drawn off and snap-frozen at −80 °C. The samples were then shipped on dry ice to Myriad RBM (Austin, TX, USA) for performance of the bead-based quantitaive immunoassay for 68 analytes of the RodentMAP v. 1.0 panel (Supplementary Table S1). For samples in which the analyte in question was below the laboratory’s lower threshold for accurate measurement (or, as it was termed, the “least detectable dose”) and was reported as “low”, we substituted a dummy value of 50% of the lower threshold value. For instance, if the reported least detectable dose was 68 and a particular result interpretation was “low”, we substituted “34” (i.e., half the least detectable dose) for this particular analyte for that sample.

### 2.9. Iron Assay

The quantity of ferrous and ferric ions in the spleen was determined colorimetrically at optical density 593 nm with Ferene S of Biovision kit K390-100 (Biovision, Milpitas, CA, USA). Results were expressed as nmol iron per g of spleen mass.

### 2.10. In Vitro Blood Cell Aggregation Assay

In wells of round-bottomed 96-well polystyrene microtiter plates 5 μL volumes of heparinized blood from uninfected mice were mixed with equal volumes of plasma from infected mice taken on day 5 after i.p. inoculation on day 0. Bacterial densities in the plasma were determined by phase microscopy, as described above, and quantitative PCR, as described below. The infected plasma was either untreated, centrifuged at 9500× *g* for 5 min at 22 °C, or centrifuged and then heated in a water bath at 56 °C for 30 min to inactivate complement. The plates were covered and then incubated at 37 °C for 1 h. The plates were then placed at 4 °C for 12 h. The plates were backlit on a light table and digital pictures were taken with a Nikon D5000 and AF-S Micro Nikor 60 mm lens. TIFF-format photo files were subjected to image analysis with ImageJ v. 1.34 software (National Institutes of Health). Dispersion of the blood cells in the well was measured as the area under the curve of the histogram of values above the background. Samples from wells were examined by phase microscopy at 400× magnification, and pictures were taken with QImaging’s (Surrey, BC, Canada) QIClick CCD camera.

### 2.11. Nucleic Acid Extraction and cDNA Synthesis

DNA was extracted from 20 μL of whole blood with Qiagen’s QIAmp DNA micro kit or DNeasy Blood and Tissue kit. DNA was extracted from 100 mg spleen with the DNeasy kit. In some experiments the DNA Clean & Concentrator kit (Zymo Research) was used to remove inhibitors from the DNA sample per manufacturer’s recommendations. The DNA standard for quantitative PCR was total genomic DNA extracted from culture-grown strain HS1 using a phenol-chloroform protocol as described (58). Total RNA was isolated from whole blood with TriReagent BD (Molecular Research Center) according to the manufacturer’s instructions. Nucleic acid concentrations were measured with a NanoDrop™ 1000 spectrophotometer (Thermo Scientific). RNA extracts were treated with RNase-free DNase I (Ambion) according to the manufacturer’s instructions. Reverse transcription reactions were carried out in reactions containing 2.5 μM of random hexamers (New England Biolabs), two units of Moloney Murine Leukemia Virus reverse transcriptase (New England Biolabs) per μL, 0.5 mM dNTPs, and 2 units of RNase inhibitor (Roche Applied Science) per μL in the following buffer: 5 mM Tris HCl, pH 8.3-7.5 mM KCl-0.3 mM MgCl_2_-2.1 mM dithiothreitol. Reactions were serially incubated in water baths at 25°C for 10 min, 37 °C for 3 h, and 100 °C for 5 min, and then immediately frozen at −80 °C. Reactions without the reverse transcriptase were carried out in parallel.

### 2.12. Quantitative PCR (qPCR)

The primers and probe targeted the single copy 16S ribosomal RNA (*rrs*) gene of *B. hermsii* and other relapsing fever group species as described [65,66]. The master mix was from Eurogentec (San Diego, CA, USA), and the reactions were run on either an iCycler (Bio-Rad Laboratories, Richmond, CA, USA) or a Rotor Gene 3000 (Corbett Research, Australia). By both methods the sensitivity was two to four gene copies; the coefficient of determination (*R^2^*) for replicate qPCR assays of the same samples was ≥0.90. One copy of a complete genome is equivalent to one copy of the *rrs* gene (Genbank CP000048). Quantitation of cDNA of transcripts of the *flaB* flagellin gene used the forward and reverse primers 5′-GTTGATTTCATCTGTAAGTTGCTCAATT-3′ and 5′-ACTTGCTGTTCAATCTGGTAATGG-3′, respectively, and the minor groove binding probe 6FAM-5′AACCTCTGTCTGCATC3′.

### 2.13. Growth Rate Determinations

The generation time in hours based on change in measurements of numbers (*N*) in genome copies between two time points, 0 and *t*, for the same mouse was calculated with this formula: ln(2)*(*t* – 0)/ln(*N_t_*/*N*_0_). The generation time in hours based on change in measurements of numbers in genome or RNA copies for groups of mice sampled at times into the infection was determined by linear regression of log-transformed data.

### 2.14. Statistics

Standard statistical tests were carried out with the SYSTAT v. 13 suite of programs (Systat Software, Inc., Chicago, IL, USA) and with Confidence Interval Analysis v. 2.1.2 (CIA Software, University of Southampton, Southampton, UK). Mean values and differences in means are presented with 95% confidence intervals (CIs). Parametric (*t* test) and non-parametric (Mann-Whitney rank sum) significance tests were carried out for continuous data and were two-tailed. Fisher’s exact test was used for categorical data in a 2 × 2 contingency table and was two-tailed. Linear regression was by the least-squares method, and 95% confidence interval for the slope was calculated. *Z* scores were the number of standard deviations above or below the mean of the control group values. Euclidean distance cluster analysis was performed and a two-color graphic display (heat map) of multiplex continuous data was generated using the MultiExperiment Viewer v 4.0 software (The Institute for Genomic Research, Baltimore, MD, USA) [67].

## 3. Results and Discussion

### 3.1. Growth of B. hermsii in the Blood

#### 3.1.1. Background

Novy and Knapp in their 1906 article noted “rapid multiplication” of what was likely *B. turicatae* in rats and mice and that the organism reached its maximum in the blood within three days after passage [14]. They wrote that “white mice are very susceptible” to infection, had up to four relapses, and “so regularly do [the relapses] return that one can quite accurately predict when this will occur”. They observed further that “…the number of spirilla which appear in the blood during a relapse is much less than in the first attack, which clearly indicates the existence of a partial immunity”. From the results provided in the article for the amount of spirochetes in the blood, the generation or doubling time for this *Borrelia* species in a rat was between 4 and 8 h.

Helen Russell reported in 1931 that there were lower numbers of spirochetes in the blood in relapses than the initial bacteremia in the Gambian giant pouched rat (*Cricetomys gambianus*) and that the blood was infectious for other rodents when not detectable microscopically [68]. Eidmann et al. estimated a generation time of 6 h for *B. crocidurae* in the blood of white mice [69]. Coffey and Eveland passed a *B. hermsii* isolate from the Sierra Nevada Mountains in outbred mice every 48 h before injection into Sprague-Dawley rats for their experiments [42,43]. In the rats the generation time was estimated as ~6 h. Stoenner et al. counted spirochetes under the microscope and reported a generation or doubling time for *B. hermsii* in the blood of about 3 h [47]. The doubling time of *B. turicatae* was between 6 and 7 h in mice [70] and ~7 h in vitro [71]. The doubling time was found to be similar regardless of whether mice were examined individually or if all the mice were grouped together as a whole.

**Figure 1:**
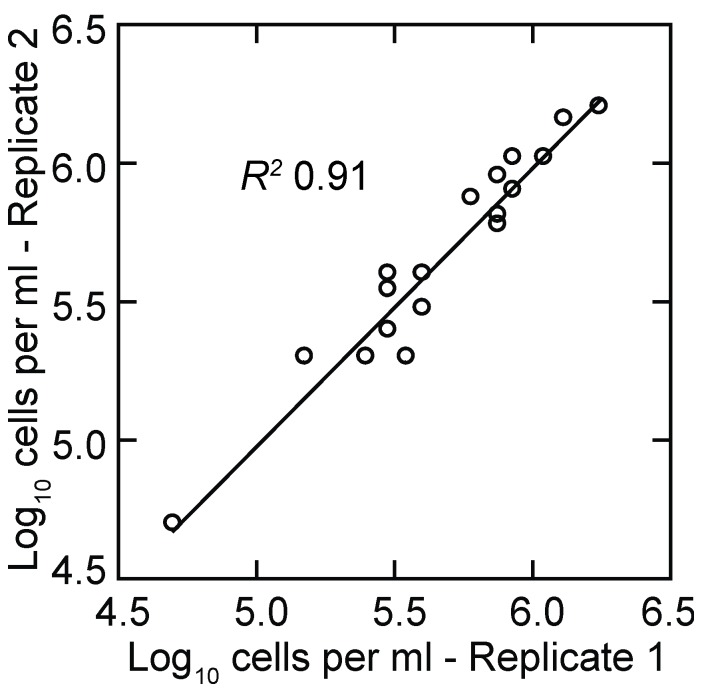
Scatter plot of replicate log-transformed counts of *Borrelia hermsii* cells in plasma samples from infected mice by phase microscopy and counting chamber. The linear regression and coefficient of determination (*R^2^*) are shown.

The ability to use a group of mice and consider them as a single animal for data analysis allows for more flexibility in experimental design since the researcher is not necessarily limited by the amount of blood in a single animal. However, counts based on microscopy are limited by the sensitivity that can be achieved with a phase or dark field microscope and a counting chamber. In our hands the detection limit for counts that use the entire counting area of the chamber is a density of ~10^4^ cells per milliliter. Benoit et al. sampled small volumes of blood and diluted it 10-fold before counting and reported a limit of detection of 10^5^ bacteria/mL [72]. Counts of replicate samplings of the same population at the extreme of sensitivity approximate a Poisson distribution, and the coefficient of variation increases as the mean approaches zero. For the present study the lower limit of measurements that provided reproducible growth rate determinations by microscopy was ~5 × 10^4^ cells/mL (Figure 1), which is what Coffey and Eveland reported for their studies of infections in rats [42].

For studies of many types of bacteria, an alternative to microscopic counts of cells is plating of non-aggregated cells on solid media and the subsequent enumeration of colonies. While this is possible with some strains of *B. burgdorferi* [73], colony formation has yet to be achieved with wild-type *B. hermsii* (unpublished findings). In addition, the practical limit for counting by plating is 10–100 colony-forming units per milliliter, depending on the number of plates used.

#### 3.1.2. PCR Quantitation of Bacteria in the Blood

**Figure 2:**
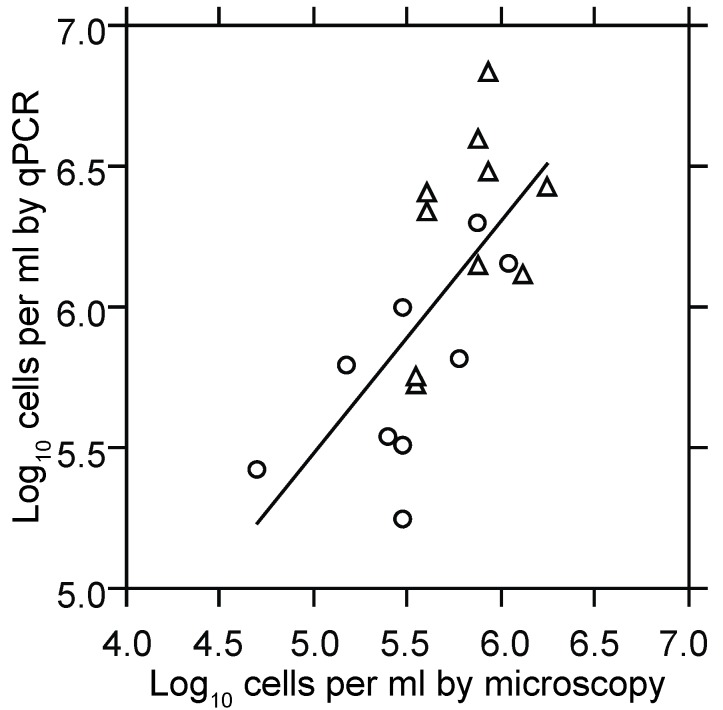
Scatter plot with linear regression of log-transformed counts of *B. hermsii* cells in plasma by phase microscopy and counting chamber (*x*-axis) and quantitative PCR (qPCR; *y*-axis). Circles and triangles denote samples of blood taken at hours 94.5 or 102 from immunocompetent BALB/c mice infected at hour 0, as described in the text. The slope (95% confidence interval) for the least-squares regression was 0.85 (0.4–1.3), and the combined *R^2^* was 0.46 (*p* < 0.001).

Quantitative PCR was the means to estimate cells numbers at low densities in the blood and thereby to measure growth of the population over a greater range. Using as a standard the total DNA extracted from a known number of cells, we reproducibly detected two to four genome copies in a PCR reaction. Given a genome copy number in the range of 10–20 per cell of *B. hermsii* [74] and the sampling volume of 20 µL of blood, the lower limit of detection of the qPCR assay used in this study was approximately five spirochetes per milliliter of blood. Figure 2 shows the results of two experiments that compared the cell counts as determined by microscopy with genome counts as determined by qPCR over a range of ~50,000 to 2,000,000 bacteria per milliliter. There were, as expected [74], 10–20 genomes measured by PCR for every cell counted by microscopy.

With this qPCR assay*,* we assessed the growth rate of serotype 7 of *B. hermsii* HS1 in the *M. musculus* host. We used BALB/c mice and their congenic SCID varieties, because of the long history of studies with this strain [75], and our experience that mice of the C57BL/6 lineage were more resistant to initial infection with *B. hermsii* than BALB/c mice (Alan G. Barbour, unpublished findings). For the experiment, 95 adult BALB/c mice were infected with one to 10 spirochetes in cohorts of 50 and 45 animals, the second cohort being inoculated 11.5 h after the first. Starting 12 h post-inoculation, a sample of blood was collected from the saphenous vein and a second sample was collected 7.5 h later. A new mouse was successively processed in this fashion every 3 h. Each mouse was bled no more than two times. DNA was extracted from each sample, quantitated, and then subjected to qPCR.

**Figure 3:**
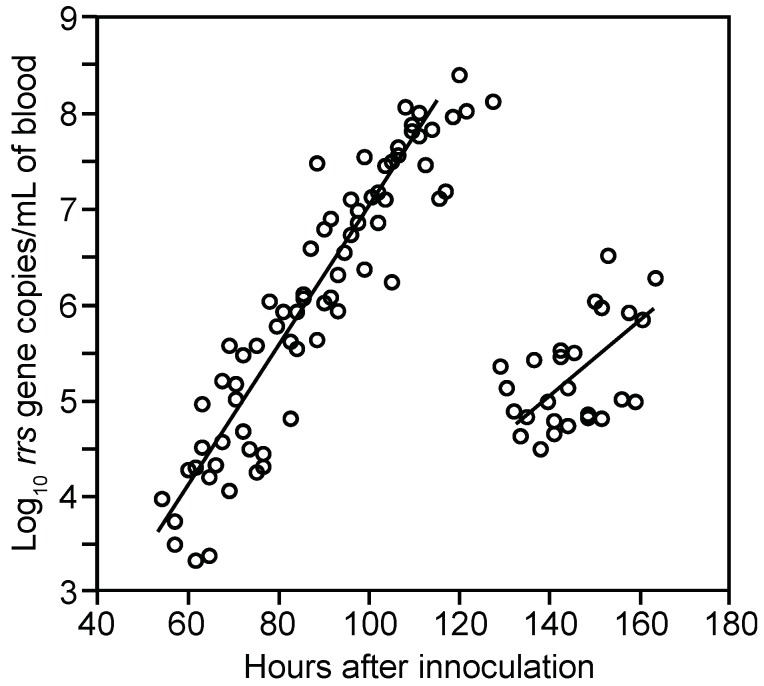
Proliferation and decline of *B. hermsii* HS1 cells in the blood of infected immunocompetent mice over time. The graph is a scatter plot of collected data on gene copies of 16S ribosomal RNA (*rrs*) of *B. hermsii* by qPCR (*y*-axis) against time in hours (*x*-axis) after inoculation of 95 BALB/c mice with serotype 7 of strain HS1. Samples were collected every 3 h beginning 12 h after inoculation; second samples were obtained from each mouse 7.5 h after the first. Each data point is a sampling from a single mouse. Linear regression determinations were made separately for hours 50–120 (*R^2^* = 0.86) and >120.

Spirochete genomes were first detected in the blood 54 h post-inoculation and thereafter displayed steady growth up to around 120 h, at which time spirochete genome densities were as high as 1.2 × 10^8^ per milliliter (Figure 3). This density corresponded to ~10^7^ cells per milliliter at the peak in these immunocompetent mice. In comparison, for three BALB/c SCID mice infected with the same serotype, the mean peak density was six-fold higher, at 7.5 (4.7–10.0) × 10^8^ genomes per milliliter of blood.

**Figure 4:**
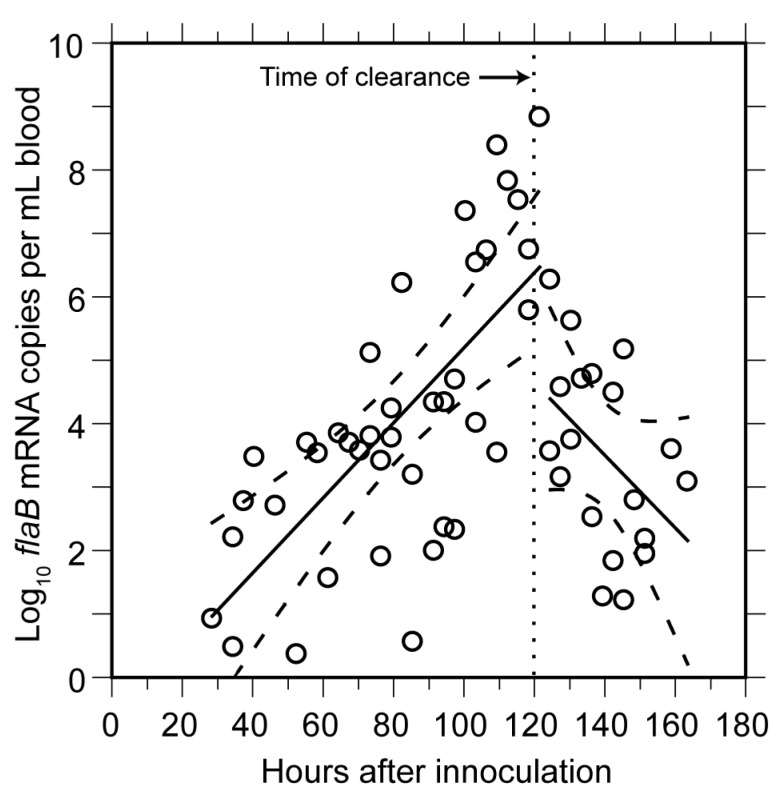
Rise and decline in mRNA for FlaB flagellin of *B. hermsii* in blood of infected mice. The experiment and sampling protocol were the same as described for Figure 3. The *flaB* transcripts were estimated by qPCR of cDNA produced with random hexamers. Each data point is a sampling from a single mouse. Linear regression determinations (with 95% confidence intervals denoted by dashed lines) were made separately for time points up to hour 120 and after hour 120. The time of clearance indicated is the approximate time point that genome counts began to decline (Figure 3).

The means and 95% confidence intervals for the doubling time of genomes by linear regression for each of the two cohorts were calculated separately to stratify for possible diurnal differences in in vivo growth. The first and second inoculation cohorts had generation times of 4.5 (4.0–5.1) h and 4.1 (3.6–4.8) h, respectively, for the period when *B. hermsii* DNA was first detected to peak. We also calculated generation times separately for measurements of first samplings and second samplings to assess possible effects of a prior bleeding of the mouse. The mean generation times for these two sets were similar: 4.5 (4.0–5.1) h and 4.2 (3.7–4.9) h, respectively. The overall genome doubling time for 69 samples obtained from hours 54 to 120 was 4.1 (3.9–4.6) h with an *R^2^* value of 0.86 (Figure 3). A second estimate of the growth rate was obtained by averaging generation times individually calculated from pairs of counts for 23 mice that were sampled between hours 70 and 110 of infection. There was greater variance in the estimate based on pairs, but the generation time of 4.8 (3.8–6.6) h was similar to that estimated by linear regression for the mice as a group. The increase in copies of DNA in the blood was paralleled by the increase in the mRNA for the *flaB* flagellin gene, which is constitutively expressed in vivo and in vitro [76] (Figure 4). The doubling rate for *flaB* mRNAs at 5.1 (3.8–7.7) h was similar to that of the DNA. 

#### 3.1.3. Clearance of Bacteria from Blood

Beginning around hour 120 (or after five days), there was a steep decline in the genome density in the blood over a period of 6 h (Figure 3). At the nadir there were 2.5 × 10^4^ genomes, or about 1300–2500 bacterial cells, per milliliter of blood. This was below the usual threshold for detection of cells by microscopy of unconcentrated blood. The decline had an estimated half-life of 1.4 h for the collective samples, but for individual mice the clearance of 99% or more of the cells from the blood could have occurred more rapidly. A slower decline in concentration was noted for the *flaB* transcripts in the whole blood (Figure 4).

#### 3.1.4. Relapse of Bacteria in the Blood

From this nadir in *B. hermsii* genomes, the counts rose again logarithmically during what we designated as the relapse. This relapse population was identified by the sequence of the expressed gene for a variable major protein which as predominantly serotype 26, which was previously noted to commonly follow serotype 7 in infections of mice [77]. The generation time for the relapse population, as determined by overall linear regression was 7.6 (4.6–22) h, slower than that for infecting serotype 7.

In a previous study we had not observed a difference in growth rates in vivo between two serotypes, 7 and 19 [78]. So, the slower growth rate of serotype 26 in the relapse population than serotype 7 in the original infecting population was unexpected. To determine whether serotypes 7 and 26 inherently grow at different rates, serotype 26 of this specific relapse was first obtained in a pure population by cloning by limiting dilution in mice. This clonal population was then in turn compared with serotype 7 in naïve mice. Groups of eight six-week-old female BALB/c mice were infected with one to five cells of either serotype 7 or 26 from the plasma of infected SCID mice. Blood samples that were taken from each of the mice 57 and 74 h after inoculation were subjected to DNA extractions and qPCR for quantitation of genome copies. The growth rates were calculated for each of the mice from pairs of data. Serotype 7 in this set of mice had a mean genome generation time of 4.4 (3.8–5.3) h. Serotype 26 in naïve mice had a generation time of 5.3 (4.5–6.5) h. This was not significantly different from that of serotype 7 (*p* = 0.4), but was faster than what was observed for the relapse population.

#### 3.1.5. Comments

An advantage of qPCR over microscopy for measuring growth is its sensitivity for low numbers of gene copies in the reaction. A drawback of qPCR is its endpoint of genomes of cells and not cells per se. *Borrelia* cells with their filamentous shapes have multiple genomes per individual cell [74]. Equating genomes with cells gives unrealistically high estimates of cell densities in the blood [79]. As the stationary phase is approached, the lengths of cells increase, and there may be correspondingly more genomes per cell, thus confounding inferences of cell numbers from genome quantities. This may occur in SCID mice if the stationary phase of growth is reached for the bacteria in the absence of immunity. However, we found that the peak densities in immunocompetent mice before clearance were five- to 10-fold lower than what we observed in the SCID mice here and what others reported for immunodeficient mice [79,80]. Thus, it is likely that the spirochetes were in the log-phase of growth in the blood of BALB/c mice until antibody-mediated clearance began on day 5.

Benoit et al. in their study of three different inbred strains of mice reported that juvenile BALB/c mice were more “resistant” to *B. hermsii* infection than either C57BL/6 or C3H/HeJ mice were [72]. This conclusion was based on their observation of a four- to six-fold difference between the strains in peak spirochete counts in the blood of the mice. In the experiment they used an inoculum of 10^5^ bacteria from a broth culture and delivered it intravenously. Our experience is that rodents can be infected routinely with one to five organisms [47,78,81], as has been similarly found by investigators of *B. duttonii* [82] and *B. turicatae* [83]. In the Benoit et al. report, the peak of spirochetemia occurred on day 3, which would be unexpected for an infection initiated by a tick bite or a much lower needle inoculum [72]. There was not a significant difference between the three strains in their counts on day 2. The relatively depressed spirochete count in BALB/c mice on day 3 was as plausibly attributable to an earlier antibody response in this strain as to a difference in innate immunity.

In the experiment with 95 mice, following the clearance event during day 5, an estimated 1000 spirochetes per mL of blood remained. This is consistent with previous reports of persistent infectiousness of blood between relapses [14,68,84]. Assuming an antigen switching rate of ~10^−4^ per cell per generation for strain HS1 [47], we would expect a relapse population size of ~3000 spirochetes by the time the total bacterial density reached 10^7^ per milliliter of blood. So, our experimental finding was about what we anticipated.

During the period of relapse, the overall population increased at a slower rate than did the infecting serotype 7 during its run-up in the blood. However, in immunologically naïve mice, serotype 26, which was the predominant serotype of the relapse population, had a similar growth rate to that of serotype 7. This finding is consistent with Coffey and Eveland’s observation that each relapse had successively lower peak densities in their rats infected with *B. hermsii*, while in naïve animals the different serotypes of relapses grew just as fast as the initiating serotype [42]. In trypanosomes, another blood-borne pathogen that undergoes antigenic variation, the expressed antigenic protein does not appear to be a major determinant of the growth rate of the organism [85].

### 3.2. Innate Host Responses to B. hermsii Infection

#### 3.2.1. Background

The last experiment’s results and our review of the literature indicated that the slower growth rate of the relapse population was attributable to host factors and not to inherent differences between serotypes in growth rates. Since the proliferating relapse population presumably was unaffected by the circulating antibodies that were specific for serotype 7 cells [42,47], the slower growth rate and lower peak densities plausibly were attributable to non-adaptive, or innate, host responses to the infection. Benoit et al. observed a temporary decline in the numbers of spirochetes after the initial peak in the blood of B cell-deficient *rag2*^−/−^ mice, a finding which was interpreted as evidence of a role of innate immunity in reducing the bacterial burden [72].

Taken together, these findings indicate that innate immunity, as it develops in response to the infection, can restrain spirochete proliferation. However, by what mechanism(s) does this occur? Innate immunity during relapsing fever remains poorly understood. Perhaps the best known manifestation of these responses and the one with major clinical significance is the Jarisch-Herxheimer (J-H) reaction, the shock-like state that occurs within a few hours of the initiation of antibiotic therapy of patients [86]. This state is associated with marked elevations of pro-inflammatory cytokines [87]. At one time this phenomenon was attributed to an “endotoxin” released by the dying spirochetes [88]. Neither *Borrelia* spp. nor *Treponema pallidum*, another agent associated with the J-H reaction, have lipopolysaccharides with endotoxin-like activity [89,90]. More likely, the so-called “cytokine storm” is in response to the release from distressed or autolyzing cells of large numbers of membrane blebs bearing abundant lipoproteins [91]. The bacterial lipoproteins are recognized by Toll-like receptors (TLR), principally TLR2, and a signaling cascade leading to the release of pro-inflammatory cytokines and chemokines follow [92]. This in turn elicits an anti-inflammatory counter-reaction, which is exemplified by the rise in cytokine IL-10 levels [93,94].

#### 3.2.2. Analysis of Proteins in Plasma during Experimental Infection

To begin to identify innate mechanisms for the restraint on spirochete growth by day 5 of infection, we examined the concentrations of various cytokines, chemokines, acute phase reactants, or other serum components at different stages of infection and compared those values to those of uninfected controls measured at the same time. To this end, 15 BALB/c mice were inoculated with an estimated one to two spirochetes of serotype 7 of strain HS1, and five mice were inoculated with the same volume of PBS as non-infected controls. The mice were then monitored for the presence and density of spirochetes by microscopic examination of tail blood. Of the 15 inoculated mice, 12 became infected, and these mice were the source of the plasma for the subsequent studies. At the peak of bacteremia on day 4, anti-coagulated blood was collected from six mice by terminal exsanguination under anesthesia. On day 5 there were no microscopically detectable spirochetes in the blood of the remaining six infected mice, and anticoagulated blood was collected by terminal exsanguination from these six mice. The three inoculated but uninfected mice and the five control mice were likewise terminally exsanguinated on day 5.

Individual plasma samples were analyzed for a concentration of 68 different proteins in the blood by immunoassay with specific antibodies (Table S1). A preliminary analysis of the data showed no significant difference between the three inoculated but uninfected mice and the five mice injected with buffer alone, so these were combined into a group of eight that was categorized as the control of “uninfected”. The other two groups for the analysis were “peak” of infection (day 4), and “clearance” of infection (day 5). At the time of the peak, the mice typically had ruffled fur and reduced locomotor activity in comparison to uninfected animals. There was no evidence of the neurologic disorders we had noted in mice infected with *B. turicatae* [71].

**Figure 5:**
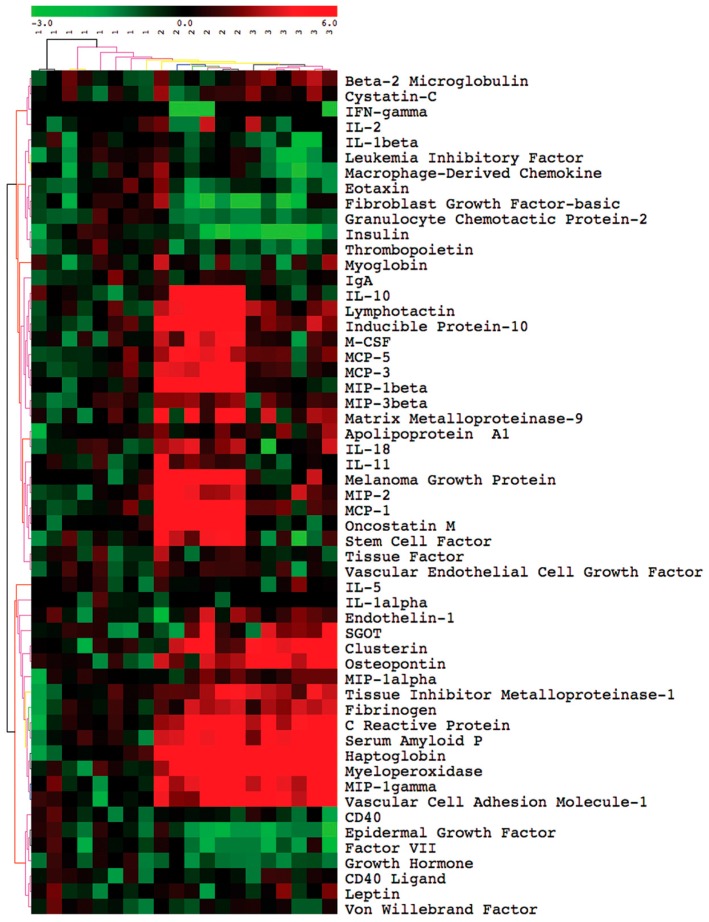
Cytokines, chemokines, acute-phase reactant, and selected other serum proteins in plasma of BALB/c mice before (group 1), at the peak of spirochetemia (group 2), and after clearance (group 3) of infection with *B. hermsii*. The figure is a two-color display and gradient heat map of values (normalized across the individual assays by *Z* scores) ranging from −3 (green) to +6 (red) for 20 plasma samples and 68 analytes, which were assayed by bead-based immunoassays, as described in the text. The analytes and their abbreviations and alternative names are listed in Table S1. Differences in means between time points and conditions 1, 2, and 3 for 39 analytes are given in Table 1.

Figure 5 is a two-color graphical representation in heat map format of the results of the analysis with the 68 analytes and the three groups of samples: uninfected (designated as “1” in the figure), peak (“2”), and clearance (“3”). The data were normalized across the different analytes as *Z* scores, which were within a range of −3 to +6 and corresponded to numbers of standard deviations below or above the mean of the controls. The heat map is structured horizontally by experimental group and vertically by similarities in profiles. Three general patterns of analyte results across the three groups are apparent: similar analyte levels for all conditions, elevated values during the peak of infection and a decline with clearance, and elevated values over the controls during both the peak and clearance. The 28 analytes of Table S1 that did not significantly vary at the 0.05 level by either parametric or non-parametric tests between the three conditions were the following: apoliprotein A1, calbindin, CD40, CD40 ligand, cystatin, eotaxin, Factor VII, Fibroblast Growth Factor (FGF)-9, FGF-2, the chemokine Granulocyte Chemotactic Protein-2 (CXCL6), Granulocyte Macrophage-Colony Stimulating Factor, glutathione *S*-transferase, Interferon (IFN)-γ, Interleukin (IL)-1α, immunoglobulin A, IL-2, IL-3, IL-4, IL-12, IL-17, leptin, Leukemia Inhibitory Factor, myoglobin, lipocalin-2, RANTES, Tissue Factor, thrombopoietin, and von Willebrand Factor. 

These three patterns were also observed when differences in means between the pairings of the three different conditions, e.g., peak vs. uninfected, etc., were calculated (Table 1). In addition, two other patterns were noted among the group of analytes that were not elevated at the peak of spirochete density: first, elevation of an analyte only at the time of clearance of infected mice, and, second, reduced concentrations of a serum protein in the infected mice, either at the peak or clearance. Table 1 lists 39 analytes that differed between at least two of the conditions, as well as, for comparison, three other analytes (IFN-γ, IL-1α, and IL-2) that showed no discernible difference from uninfected controls.

The serum proteins that were significantly elevated at the peak but had declined by the time of clearance were following: (i) the cytokines IL-6, IL-7, IL-10, IL-11, IL-18, Macrophage-Colony Stimulating Factor (M-CSF), Oncostatin M, Stem Cell Factor, and Tumor Necrosis Factor-α (TNF-α); (ii) the chemokines Inducible Protein-10 (CXCL10), KC/GROα (CXCL1), lymphotactin (XCL1), Monocyte Chemoattractant Protein (MCP)-1 (CCL2), MCP-3 (CCL7), MCP-5 (CCL12), Macrophage Inflammatory Protein (MIP)-1β (CCL4), and MIP-3 β (CCL19); (iii) the enzyme Matrix Metalloproteinase-9 (MMP-9), and (iv) the growth factor Vascular Endothelial Cell Growth Factor (VEGF).

The serum proteins that were significantly elevated at the peak and remained so after clearance were the following: (i) the chemokines MIP-1α (CCL3) and MIP-2 (CXCL1); (ii) the acute-phase proteins C-reactive Protein (CRP), fibrinogen, haptoglobin, and Serum Amyloid P (SAP); and (iii) clusterin, myeloperoxidase, osteopontin, Tissue Inhibitor of Metalloproteinase-1 (TIMP-1), and Vascular Cell Adhesion Molecule-1 (VCAM-1). 

Analytes that were higher at the clearance sampling but not at the peak were the vasoconstrictor endothelin-1, the MHC class I component β-2 microglobulin, and the liver enzyme serum glutamic-oxaloacetic transaminase (SGOT). Significantly lower concentrations at either the peak or clearance samplings than in uninfected mice were observed for Epidermal Growth Factor (EGF), the cytokine IL-1β, Macrophage-Derived Chemokine (CCL22), and the hormones insulin and growth hormone.

The profiles of these analytes in the infected immunocompetent mice were similar to what we observed with SCID mice of the same genetic background infected with the CC1 strain instead of the HS1 strain [95]. Infected mice at peak spirochete densities had 10- to 20-fold higher concentrations in comparison to uninfected mice of both the pro-inflammatory cytokine IL-6 and the anti-inflammatory cytokine IL-10, as well as the inflammation chemokines MIP-1α (CCL3), MIP-1β (CCL4), MIP-2 (CXCL1), MCP-1 (CCL2), MCP-3 (CCL7), and MCP-5 (CCL12). Other serum components that were elevated in both infected SCID and infected immunocompetent mice were the acute-phase proteins CRP, haptoglobin, and SAP, as well as endothelin-1, myeloperoxidase, MMP-9, TIMP-1, and chemokine MCP-5 (CCL12). As we observed with infected immunocompetent mice, there were no discernible elevations of either cytokine IFN-γ nor IL-1β in the infected SCID mice [95].

#### 3.2.3. Comments

Overall, the profile of the analyte changes in the infected immunocompetent mice corresponds to a marked systemic inflammatory response and an accompanying modulation of those pro-inflammatory cytokines and chemokines by anti-inflammatory cytokines, such as IL-10. There were several similarities to and some differences from the early responses of mice to injections of *E. coli* lipopolysaccharide (LPS) [96]. More specifically, 4 h after injection the LPS-treated mice showed marked elevations of both IL-6 and IL-10, and while IL-6 had declined toward normal by 24 h after injection, IL-10 remained elevated. The concentration of chemokines MIP-1β, MIP-2, MCP-1, MCP-3, and MCP-5 at 4 h after LPS injection were comparable to those of *B. hermsii*-infected mice. A limitation of the study was the absence of the B cell chemokine CXCL13 from the analyte panel. Gelderblom et al. reported high CXCL13 concentrations in the blood of B cell-deficient C57BL/6-*Igh6*^-/-^ mice infected with *B. turicatae* [97]. 

### 3.3. Antibody Responses to B. hermsii Infection

#### 3.3.1. Background

In 1896 Gabritchewsky reported that serum from a recovering relapsing fever patient lysed the etiologic spirochetes when they were mixed together in the laboratory [98]. In 1906 Novy and Knapp showed that once infected animals had recovered, they were immune to challenge by the same organism and, in addition, that anti-serum obtained from the immune mice provided passive protection for other animals against infection [14]. Novy and Knapp noted that the antibodies were both agglutinating and bactericidal. Stoenner et al. and Barbour et al. working with the *B. hermsii* model of mice demonstrated that anti-serum to a specific serotype would clear the blood of that particular serotype but not other serotypes [47,99]. Newman and Johnson found that neither T cells nor the terminal components of complement were necessary for clearance of *B. turicatae* from the blood of mice [100,101]. IgM antibody is sufficient for clearance from the blood [99,102]. Some of the antibodies elicited by infection or immunization are bactericidal in the absence of a complement [99,103,104].

#### 3.3.2. Agglutination Assays

**Figure 6:**
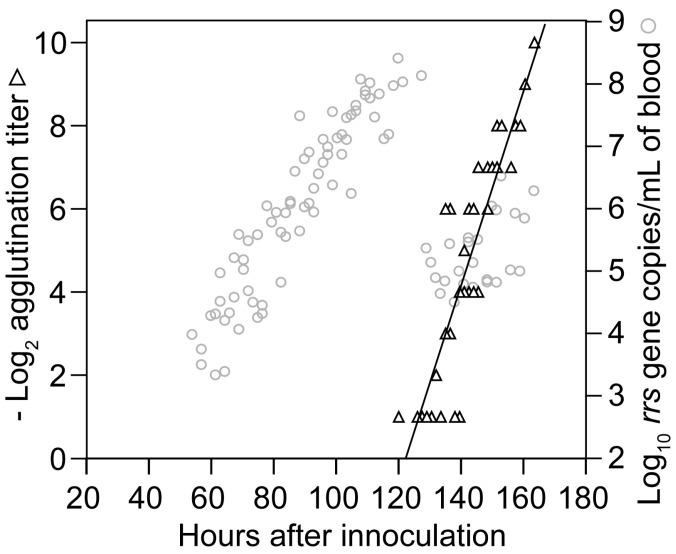
Rise in agglutinating antibodies to *B. hermsii* during infection. The graph superimposes a scatter plot of log-transformed reciprocal titers of antibody to serotype 7 of *B. hermsii* by a macro-agglutination assay (black triangles) on to the values for *B. hermsii* growth in the blood (gray circles) of Figure 3. The samples were drawn from the same group of mice described for Figure 3 and Figure 4 (Section 3.1.2). Each data point is a sampling from a single mouse.

Assays for antibody function, such as agglutination, neutralization, or growth inhibition, are more predictive of protection against infectious challenge by *Borrelia* spp. than matrix-based assays, such as ELISA or Western blot [105,106]. Accordingly, we examined by macro-agglutination assay the plasma collected during the course of the growth experiment with 95 mice that is described in Section 3.1.2. The antibody to serotype 7 was first detected by agglutination at about the same time as the sharp decline in spirochetes in the blood began at hour 120 (Figure 6). The agglutinating antibody concentration doubled every 4.3 (3.6–5.3) h for the duration of the experiment. This rate was similar to the genome doubling rate for *B. hermsii* in the blood.

To assess the specificity of these agglutinating antibodies, on day 0 we infected groups of five BALB/c mice with an estimated one to two spirochetes of either serotype 7 or serotype 19, which had been propagated in SCID mice. On day 5 anti-coagulated blood was obtained from all mice by terminal exsanguination. Whole blood was subjected to qPCR: four of five serotype 7-injected mice were positive, and three of five serotype 19-injected mice were positive. The genome count per milliliter of blood in the infected mice ranged between 138 and 16,600 with a mean of 3811, approximately the same genome density observed at the time of clearance in Section 3.1.2. For the micro-agglutination assay, cell-free plasma from each mouse was then mixed 1:1 with SCID mouse plasma containing either serotype 7 or 19. All four BALB/c mice who had been infected with serotype 7 had agglutinating antibodies against serotype 7 cells but not to serotype 19 on day 5. In contrast, on day 5 all three BALB/c mice infected with serotype 19 had agglutinating antibodies to serotype 19 but not serotype 7 (*p* = 0.03). The three uninfected mice did not have detectable agglutinating antibodies to either serotype 7 or 19 cells. This experiment indicated that these early-appearing antibodies were serotype-specific, and thus permissive of the growth of other serotypes.

#### 3.3.3. Antibody Response Detected by ELISA and IFA

**Figure 7:**
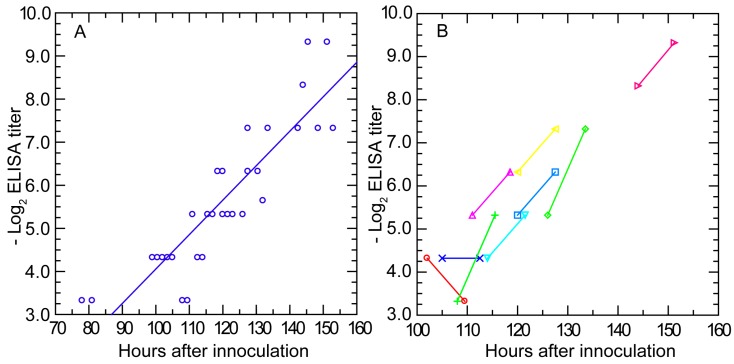
Serum antibody to whole cells of *B. hermsii* by enzyme-linked immunosorbent assay (ELISA) over time in infected mice. The experiment and blood collection protocol are the same as described for Figure 3. Panel (**A**) is a scatter plot of log-transformed reciprocal titer values for individual mice against time in hours after inoculation; Panel (**B**) shows pairs of values for nine individual mice obtained 7.5 h apart and denoted by different colors and symbols as given in the figure.

While matrix-based assays such as ELISA have lower predictive value for assessing immunity status [99,107], they often have the advantage of greater sensitivity for detection of antibodies to a given pathogen. Accordingly, we examined the antibody response to infection with serotype 7 strain HS1 using a whole-cell ELISA. Mouse serum samples collected from the 95-mouse experiment of Section 3.1.2 were assayed for the presence of antibody to serotype 7 cells. Anti-*B. hermsii* antibodies were detectable in the blood at a reciprocal titer of ≥20 at hour 99 post-infection, which was about 20 h before clearance was underway and agglutinating antibodies were first detected (Figure 7, panel A). The amount of antibodies reacting with cells in the wells increased over time with a doubling time of 9.8 (7.9–11.6) h and an *R^2^* of 0.78. By hour 120 post-infection, antibody levels were at reciprocal titers between 40 and 80.

There were nine mice for which there were pairs of ELISA results, with the first bleeding beginning at hour 100 post-infection and then with a second bleeding 7.5 h later (Figure 7, panel B). From hour 110 onwards, the anti-*B. hermsii* antibody levels approximately doubled during the testing interval, a result consistent with the doubling rate calculated by linear regression from the collected individual values of the entire group. These findings indicated that production of anti-*B. hermsii* antibodies began no later than day 4 of an infection started from fewer than 10 spirochetes. The total number of antibodies reactive against antigens displayed on the matrix increased at a rate one-half of that noted for agglutinating antibodies against live bacteria.

The early appearance and then increase of antibodies while the spirochetes were still multiplying in the blood was further documented by IFA for IgM antibodies. Five plasma samples from each of the time points at hours 66, 72, 78, 84, 94.5, 100.5, 106.5, and 112.5 into the infection were examined at a dilution of 1:10 for the first appearance of reactivity to either serotype 7 or serotype 19 in thin blood smears. No reactivity to either serotype was detected in the samples from hours 66 to 84. However, at hour 94.5, one (20%) of the five mice sampled at that time had antibodies to serotype 7 cells by IFA. The proportions of the mice with antibodies to serotype 7 thereafter increased from two (40%) of five at hour 100.5 to three (60%) of five at hour 106.5 and then to four (80%) of five at hour 112.5. No mouse had detectable antibodies to serotype 19 or any shared antigens between serotype 19 and serotype 7 before clearance had occurred. 

#### 3.3.4. Antibody Response in Nude Mice

Newman and Johnson demonstrated that nude mice deficient in T cell-mediated immunity cleared *B. turicatae* from the blood as well as congenic immunocompetent ones and within the same time [101]. We subsequently confirmed this with nude mice infected with *B. hermsii* [99]. Here we report the timing of antibodies to the infecting strain in nude mice. Five five-week-old female homozygous nude mice and three five-week-old female BALB/c mice were injected with ~10^4^ serotype 7 cells from an infected mouse on day 0. Five other nude mice were injected with buffer alone. On days 2 or 3 spirochetes were observed in tail vein blood of all the mice injected with *B. hermsii*. On day 4 no spirochetes were noted in the blood of any of the mice, and the mice were exsanguinated on that day. By IFA with sera serially diluted two-fold beginning with 1:2, we observed reciprocal titers of 16, 128, and 256 for the BALB/c mice and reciprocal titers of 16, 32, 64, 64, and 64 for the five nude mice on the same day on which clearance was first recorded (*p* = 0.4 for log-transformed data). 

#### 3.3.5. In Vitro Neutralization with a Monoclonal Antibody

The simultaneous presence of the *B. hermsii* cells and antibodies to *B. hermsii* in the blood before final clearance occurred raises the question about the relationship between densities of the spirochetes and concentrations of the antibody. The in vivo findings suggested that there may be, for both cells and antibodies, critical threshold densities and concentrations, only above which the functional effect, such as agglutination or growth inhibition, manifests. We investigated this using a growth inhibition assay, which had been shown to correlate better with protective immunity for both vaccinated mice and humans than matrix-based immunoassays, such as ELISA [105,106]. The assay usually provides an unambiguous distinction between wells in which there is unimpeded growth and those with inhibition. Different concentrations of serotype 33 cells and antibody to serotype 33 were distributed in microtiter plates in a checkerboard format [63]. The antibody was a monoclonal antibody that had both agglutinating and growth-inhibiting properties [63]. The study was carried out in the presence and absence of complement. 

**Figure 8:**
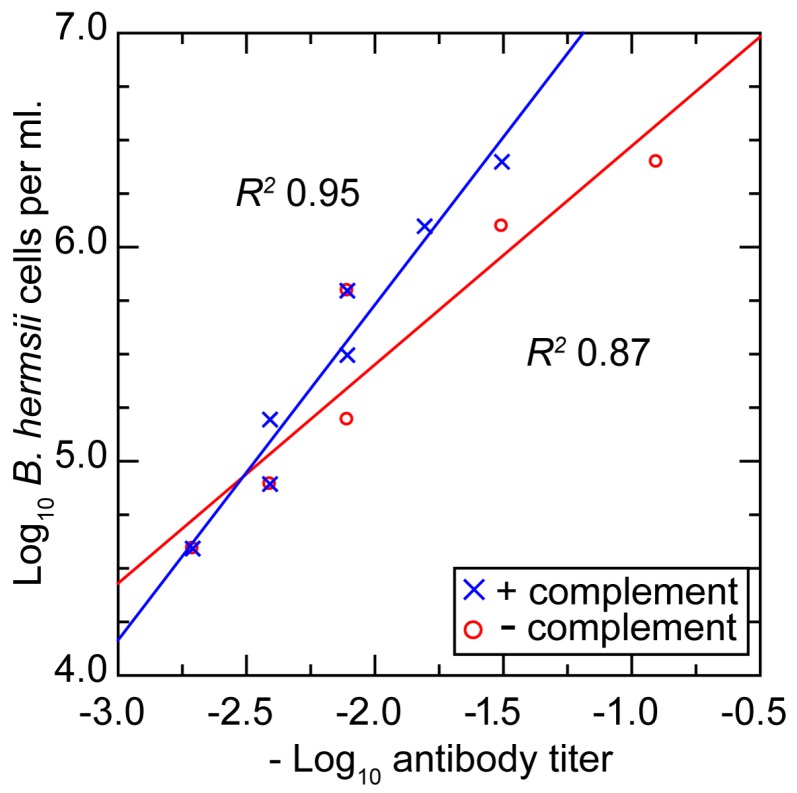
Scatter plot of in vitro growth inhibition assay titers at different densities (log-transformed) of *B. hermsii* cells and different titers (log-transformed) of a monoclonal antibody to *B. hermsii* and in presence or absence of complement. Linear regression for each of the two conditions and corresponding coefficients of determination (*R^2^*) are shown.

Figure 8 shows plots of the densities of bacteria and concentrations of antibody at which growth inhibition occurred and below which there was no discernible effect. The same ratio of antibodies to bacteria for achieving inhibition roughly held over the entire range. For a given density of cells, ~50% less antibody was required for growth inhibition when complement was included.

#### 3.3.6. Comments

Returning again to the pioneering study of Novy and Knapp [14], we noted in their rat model experiments an interval of about two days between the first detection of spirochetes in the blood and their clearance. If we assume that there were ~10^5^ bacteria per mL at detection and ~10^7^ per mL at clearance, then these kinetics of antibody production would be similar to what be observed in mice infected with *B. hermsii*. Coffey and Eveland observed the specific antibody to the infecting serotype as detected by immunofluorescence assay appeared within five days of injection of the rats [42].

Our results and those of the aforementioned studies suggest to us the following account for the simultaneous presence of both antibodies and spirochetes in the blood: Until the concentration of antibodies reaches a critical threshold, at which complete or near-complete neutralization of the targeted cells ensues, the majority of spirochetes are not affected and continue to replicate. Plausibly this is because when there are few antibodies binding to the spirochetes, they can be shed through a patching phenomenon and the release of membrane blebs or vesicles [108]. The findings suggest that the threshold for an antibody effect varies directly with the density of bacteria. The similarity in doubling rates for the spirochetes and for the agglutinating antibodies against them is noteworthy. It suggests that the head start of three days or so afforded to the pathogen over the adaptive immune response can be sustained for a few days more, until the carrying capacity of the host is reached and the rising antibody concentration finally passes the threshold.

### 3.4. Anemia during Infection

#### 3.4.1. Background

Anemia during relapsing fever occurs in human cases of the disease [109]. Anemia was noted in two of three dogs infected with *B. turicatae* [55] and in the majority of cases of *B. persica* infections in dogs and cats in Israel [57]. Anemia has also been noted in experimental infections, including *B. crocidurae* [110]. In our preliminary studies with strain CC1 of *B. hermsii* we observed decreased hematocrit values with splenomegaly in SCID mice (unpublished findings). This phenomenon was further investigated in the present study. 

#### 3.4.2. Changes in Hematocrit during Infection and after Antibiotic Treatment

In the first experiment 15 eight- to 10-week-old male C.B-17 SCID mice weighing between 19 and 23 g were inoculated on day 0 with ~10^3^ strain CC1 bacteria in freshly obtained infected mouse plasma. On day 3, infection was confirmed in all mice by phase microscopy of tail vein blood. On day 5, eight infected mice were weighed, euthanized, terminally exsanguinated, and dissected with removal of the spleen for weighing and histopathology. The seven remaining infected mice were administered ceftriaxone for three days before euthanasia and processing on day 8. At that time, four uninfected SCID mice of the same sex and age were similarly euthanized and processed.

**Figure 9:**
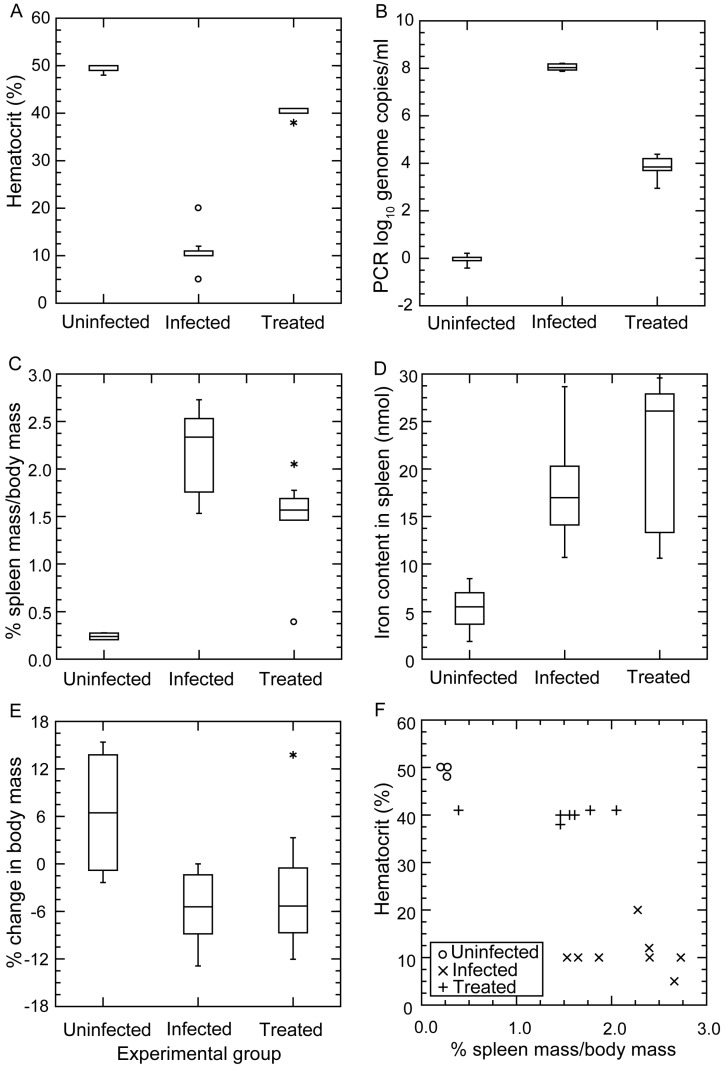
Severe anemia in BALB/c mice with severe combined immunodeficiency (SCID) and infected with strain CC1 of *B. hermsii*. The experiment is described in the accompanying text. Panels (**A**–**E**) are box-whisker plots in which each horizontal box indicates the first and third quartiles, and the indentation inside the box is the median. * The 1.5× interquartile range is indicated by the horizontal line (whiskers) bisecting the box, and values outside this range are indicated by asterisks and circles. The three conditions were uninfected (*n* = 4 mice), infected without antibiotics (*n* = 8), and infected with antibiotics (*n* = 7). Panel (**A**) is of the hematocrit values for the whole blood from the mice. Panel (**B**) is of the spirochete burdens in the groups of mice as estimated by qPCR as described for Figure 3; Panel (**C**) displays the ratios in percentages of the spleen weight to total body weight after euthanasia; Panel (**D**) shows the iron content in the spleen in nmol per gram of spleen; Panel (**E**) shows the net change in body mass in percentage between initial measurement and measurement at time of euthanasia; Panel (**F**) is a scatter plot of hematocrit values against ratio of spleen weight to body weight for individual mice in each group.

Figure 9 summarizes the results. Panel A shows the profound anemia of infected mice and the near-complete restoration of the erythrocyte count with three days of antibiotic treatment. The means (95% CI) for the hematocrits (%) of uninfected, infected, and antibiotic-treated mice were 49.5 (48.5–50.5), 10.9 (7.9–13.8), and 40.1 (39.3–41.0), respectively. The short course of antibiotics was effective in reducing the bacterial count in the blood by ~99.99% (panel B). However, while the red blood cell count increased with a decrease in the number of bacteria in the blood (panels A and B), the spleens remained enlarged (panel C). The mean percentage ratios (95% CI) of spleen weight-to-body weight for uninfected, infected, and antibiotic-treated mice were 0.24 (0.20–0.28), 2.2 (1.9–2.5), and 1.5 (1.1–1.9), respectively. The continued elevated iron content of the spleen after treatment (panel D) was an indication that the enlarged spleen was attributable to retained erythrocytes or their debris inside the spleen. The constitutional effects of the infection on the mice were seen in the weight loss displayed by the infected mice, even those who had received the antibiotic (panel E). The relationship between the hematocrit and relative spleen weight in the infected mice and the corresponding discordance in those parameters in the treated mice are shown in panel F.

#### 3.4.3. Histopathology

Microscopic examination of thin sections of fixed spleens from four infected mice and one uninfected mouse revealed sinusoids that were heavily congested with erythrocytes in the infected mice. There was erythrophagocytosis and marked vasculitis with neutrophils in the walls of arteries and arterioles. Hematopoietic units, with their tell-tale megakaryocytes, were present in the congested spleens of infected mice and were evidence of extramedullary hematopoiesis.

#### 3.4.4. Comments

Benoit et al. observed that the B cell-deficient *rag2*^-/-^ mutants of strains BALB/c, C57BL/6, and C3H/HeN became anemic as well as thrombocytopenic with infection of strain DAH *B. hermsii* of an unreported serotype [72]. On day 5 of infection, the erythrocyte counts were about 40% lower than what they were on day 0 [72]. The drop in red blood cells and platelets inversely correlated with spirochete burden and with spleen and liver weights, as noted here (Figure 9). Histopathologic studies by Benoit et al. revealed inflammatory cells, marked erythrophagocytosis, and extramedullary hematapoiesis in spleens of the infected immunodeficient animals [72]. 

The role of the spleen in limiting pathogen burden during spirochetemia was recognized as early as 1928, when Meleney reported a more severe course of relapsing fever in squirrels that been splenectomized [111]. This was confirmed in subsequent studies by, among others, Baltazard in 1937 [112] and Alugupalli et al. more recently [113]. While splenectomy could also affect the adaptive immune response, the greater deficiency during relapsing fever appears to result in loss of the spleen’s filtering function for spirochetes, either in aggregates with blood cells, with antibodies, or both. The release back into the circulation of most of erythrocytes with antibiotic therapy in the present study indicates that much of the erythrocyte sequestration, whether in the spleen, liver, or elsewhere, is reversible. 

Moderate to severe anemia also occurs in mice experimentally infected with *Trypanosoma brucei*, another blood-borne, extracellular pathogen that features antigenic variation [114]. This has been attributed to sensitization to IgM-antigen complexes [115], inflammatory cytokines such as TNF-α [116], and erythrophagocytosis [117].

### 3.5. Erythrocyte Aggregation during Infection

#### 3.5.1. Background

The adhesiveness of relapsing fever *Borrelia* cells for erythrocytes in the blood of infected patients was noted by Cook as early as 1904 [118]. This phenomenon of erythrocyte rossetting was explored in depth by Bergström and colleagues with the “Old World” species *B. crocidurae* of Africa [110,119]. Although an in vitro interaction of *B. hermsii* cells with human erythrocytes had been noted [120], this species has drawn most scientific interest for its cells’ interactions with platelets and the consequent thrombocytopenia [53].

#### 3.5.2. In Vitro Aggregation Experiment

The foregoing study of experimental infection suggested that the anemia was attributable in part to sequestration of the erythrocytes and, furthermore, that much of this was reversible with timely antibiotic therapy. The antibiotic presumably had this salutary effect by killing or disarming spirochetes, thereby releasing whatever blood cells had been removed from the circulation of blood. However, not all was reversible; the hematocrits rose, but not back to normal. The post-therapy persistence of splenomegaly and elevated iron in the spleen indicated that erythrocytes or their debris remained in the spleen. Since this occurred in animals lacking adaptive immunity, antibodies binding to erythrocyte-borne spirochetes in a complex could not be evoked as an explanation. 

However, it was not clear whether the sequestration was attributable to direct effects of the spirochetes, such as was noted for *B. crocidurae* and erythrocytes, or consequences of the host response. To begin to investigate this, we infected 12 adult female C.B-17 SCID mice and BALB/c mice with 10^3^ cells of strain CC1 on day 0. Infection was monitored by microscopy of tail vein blood. At or near of the peak of spirochetemia on day 5, the blood was obtained at the time of euthanasia. The pooled plasma from the infected mice was compared with pooled plasma from uninfected mice of the same strain for their aggregation effects when mixed with an equal volume of heparinized whole blood from uninfected mice of the same strain. The blood was subjected to low-speed centrifugation, brief enough to sediment blood cells but not spirochetes in the suspension. This provided infected plasma. Samples of the infected plasma were also subjected to high-speed centrifugation to pellet the spirochetes present. Some of the latter supernatants were also heated under conditions that would inactivate complement. These preparations from infected mice were compared with plasma from uninfected mice in 1:1 mixtures with heparinized whole blood from uninfected mice.

**Figure 10:**
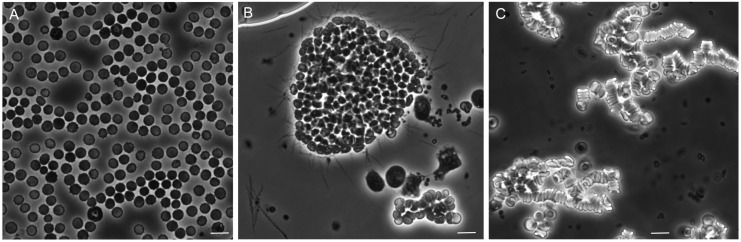
Phase photomicrographs of uninfected mouse blood mixed with equal volumes of plasma from uninfected SCID mice (panel **A**); plasma from *B. hermsii*-infected BALB/c mice (panel **B**); and plasma from infected mice that had been centrifuged (panel **C**). In panel B numerous spirochetes are seen singly, in clumps, and adhered to the aggregate of blood cells. Panel C is an example of rouleaux formation. The size marker in each panel is 10 µm.

Figure 10 shows representative photomicrographs of the mixtures of these plasma preparations after incubation with whole blood. There was little or no aggregation or clumping of the blood cells in the mixture of uninfected plasma with the whole blood. In the mixture of red cells with infected plasma, which contained an estimated 5 × 10^6^ spirochetes per milliliter, there were large clumps of erythrocytes with numerous spirochetes attached on the periphery. With the centrifuged plasma the spirochetes were absent. Aggregates of erythrocytes were noted, but these were characterized as rouleaux, with the red blood cells in stacks rather than a more disordered clump.

**Figure 11:**
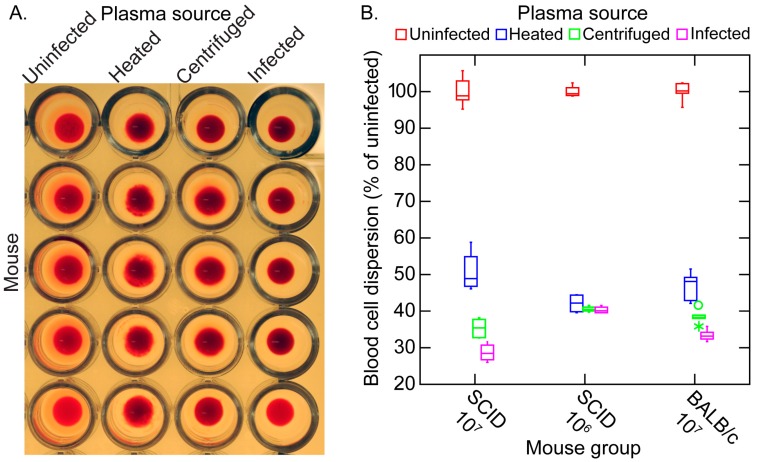
In vitro erythrocyte aggregation assay of uninfected blood mixed with plasma from uninfected mice and plasma from infected mice that was unaltered (infected), centrifuged, or centrifuged and heated. The assay method is described in the text. (**A**) Panel A shows a microtiter plate for which the source of plasma was from immunocompetent BALB/c mice (five uninfected or five infected); (**B**) Panel B is a box-whisker graph of the blood cell dispersion as estimated by image analysis and expressed as the percentage of each of four experimental conditions’ values to the mean of the uninfected values. The 12 infected SCID mice were divided into a group of eight with a high spirochete burden (10^7^ cells/mL) and a group of four with a moderate spirochete burden (10^6^ cells/mL) in the plasma. There were five infected BALB/c mice.

These effects of the different plasma preparations in the mixtures with blood cells could also be quantitated by measuring the dispersion of cells in microtiter plates after incubation. The more disperse the cells in the wells, the wider the area of opacity. This can be seen in panel A of Figure 11. The resultant image analysis values are summarized in panel B. The samples from 12 infected SCID mice were divided into two groups by the densities of the spirochetes in the blood at the time of collection. Eight SCID mice had bacteria densities of ~10^7^ cells/mL, and in the four others the densities were ~10^6^/mL. The third group for the experiment was the immunocompetent five BALB/c mice, which averaged ~10^7^ spirochetes per milliliter. The greatest amount of aggregation was observed with infected plasma of the more heavily infected SCID mice and BALB/c mice. Centrifugation and centrifugation followed by heating reduced the aggregation, as measured by this method, but did not return the values to that of the uninfected sera. The continuing presence of rouleaux in the heated plasma was confirmed by microscopy.

#### 3.5.3. Comments

The occurrence of the aggregation with the blood of both immunocompetent and SCID blood argues against a role of antibodies. The results indicate there may be at least two components to the erythrocyte aggregation phenomenon in these in vitro assays. The first is the rouleaux formation that is commonly noted during acute infections and is attributable to acute-phase proteins, particularly fibrinogen [121]. We observed high concentrations of fibrinogen and other acute-phase proteins in the blood of infected mice and their persistence even during clearance (Table 1).

The second component appears to be the binding of the spirochetes to erythrocytes and then a secondary clumping together of smaller aggregates into the larger ones that can be seen in Figure 10. This effect may be rapidly reversible with antibiotics, especially those with bactericidal activity, such as ceftriaxone [62,122]. It conceivably could also be reversed with the rise of antibodies that have bactericidal activity [103].

The degree of anemia observed in our experiments with the CC1 strain in the SCID mice was more severe, to the point of being life-threatening, than Benoit et al. reported for their *rag2*^-/-^ mice infected with the DAH strain of unreported serotype [72]. This suggests to us that strains or serotypes within a strain differ in their capacities to mediate the aggregation phenomenon. The molecular phenotype that this corresponds to remains to be determined.

## 4. Conclusions

One caveat for our and most other contemporary animal models of relapsing fever is their dependence on either the house mouse *M. musculus* or the brown rat *Rattus rattus*. Neither of these murids are a natural reservoir of a relapsing fever *Borrelia* sp. With the exceptions of *B. recurrentis* and *B. duttonii*, humans are not natural reservoirs for relapsing fever agents either. In North America humans, like dogs, cats, and some other domestic animals, are inadvertent hosts and probably evolutionary dead-ends for these zoonotic pathogens. In that light, the *M. musculus* experimental model can be justified for the insights it provides for understanding pathogenesis and immunity of relevance for human disease. However, for a more comprehensive appreciation of the ecology of these zoonoses, a case can be made for a return to experimental studies with reservoir hosts that share evolutionary histories with a *Borrelia* sp.

In the case of *B. hermsii* in western North America, suitable alternatives to *M. musculus* among natural hosts are chipmunks or squirrels [40]. However, these species are not routinely available as laboratory-reared specimens from a breeding facility. Field capture of a sufficient number of animals for experiments that include terminal exsanguination, such as those presented here, would have been prohibitive in cost and difficult to justify on an animal welfare basis. To more fully represent the diversity of host populations, there is an argument for using outbred mice, as was routinely done before the 1980s. Inbred strains are more stereotypical in their responses and only narrowly capture the diversity of wild populations. However, more heterogeneous populations, with their greater variances of values for a variable, usually call for larger sample sizes for experiments with either a continuous or categorical endpoint.

For the nearer term, the question of whether BALB/c mice or some other strain, such as C57BL/6, is the preferred lineage to use with respect to either pathogenesis or immunity of relapsing fever in a *M. musculus* model remains unresolved. With the exception of one limited study [72], there has not been a comprehensive investigation of different inbred strains of *M. musculus* infected with *B. hermsii* or other relapsing fever to the extent to which there has been for the Lyme disease agent *B. burgdorferi* [123,124,125].

Another possible limitation of the present study was infection by needle inoculation rather than tick bite. The mice in most of the experiments were infected with low doses of organisms that were comparable to those delivered by a feeding tick. The source was fresh blood of an infected mouse, so the phenotype of infecting bacteria was that expressed in the mammal [49]. However, some of the key early events of natural infections, such as at that tick bite site, were not captured by the experiments as designed. Tick transmission of infection is a more demanding experimental design and requires a functioning breeding colony of *Ornithodoros* sp. ticks, but it still is possible, as Schwan and Lopez and their colleagues have shown [126,127,128]. 

Recognition of both the limitations and the achievements of these studies and those of other investigations informs our following views for research priorities for animal models of relapsing fever: (1) assess the growth and immune response dynamics of a relapsing fever agent in a natural host. This may be most feasible for species in North America with *P. maniculatus*, which is a reservoir host for some strains of *B. hermsii* and which has a long history of successful breeding and maintenance of colonies in captivity; (2) Keeping within the *M. musuculus* model, evaluate the diversity of host responses to infection by using a mixed-stock population (e.g., Collaborative Cross mice [129,130]) that more closely approximates the diversity of wild populations. Highly multiplexed arrays and low-cost whole-genome sequencing allow stratification by genotype; (3) Initiate more experimental infections by transmission from *Ornithodoros* sp. ticks, preferably first with an inbred strain of mouse to minimize host heterogeneity, and compare them with needle inoculations; (4) Identify the factor(s) that confer(s) partial resistance to *B. hermsii*, as evidenced by the slower growth rates and lower peak densities during relapses, before neutralizing antibodies come to the fore; (5) Further characterize the overlooked phenomenon of erythrocyte aggregation by a “New World” relapsing fever *Borrelia* sp.

## Figures and Tables

**Table 1:** Changes in selected proteins in blood of BALB/c mice during course of experimental relapsing fever.

Analyte	Difference in Means (Lower, Upper 95% Confidence Limits)				
	Unit ^a^	Peak vs. Uninfected	Clearance vs. Uninfected	Clearance vs. Peak	Change ^b^
β-2 Microglobulin	µg	+0.1 (−0.2, +0.3)	*+0.4 (+0.2, +0.6)* ^c^	+0.3 (+0.1, +0.5)	*C*
Clusterin	µg	*+62 (+19, +106)*	*+131 (+91, +171)*	+69 (+7, +130)	*P,C*
C-reactive protein	µg	*+3.8 (+2.5, +5.0)*	*+3.6 (+2.3, +4.8)*	0.2 (−2.0, +1.6)	*P,C*
EGF ^d^	pg	−3.6 (−8.0, +0.7)	−*6.5 (*−*10.8,* −*2.3)*	−2.9 (−8.0, +2.2)	(P,*C*)
Endothelin-1	pg	+5.5 (−9.6, +20.5)	+11.2 (+3.3, +19.2)	+5.8 (−10.9, +22.4)	C
Fibrinogen	µg	*+3421 (+1710, +5131)*	*+3703 (+2135, +5269)*	+282 (−1643, +2207)	*P,C*
Growth hormone	ng	−15.8 (−28.2, −3.4)	−14.3 (−26.5, −2.0)	+1.5 (−3.2, +6.2)	(P,C)
Haptoglobin	µg	*+88 (+70, +105)*	*+112 (+103, +121)*	+25 (+1.7, +48)	*P,C*
IP-10 (CXCL10) ^e^	pg	*+151 (+108, +195)*	*+29 (+13, +45)*	*−122 (−173,* −*71)*	*P*
Interferon-γ	pg	+6.0 (-0.5, +12.6)	+1.3 (−1.1, +3.6)	−4.8 (−13.0, +3.5)	
IL-1α	pg	−26 (−62, +9)	−28 (−64, +7.9)	1.4 (−4.2, +1.4)	
IL-1β	pg	+120 (−670, +910)	−900 (−1810, +10)	−1020 (−1690, −350)	(C)
IL-2	pg	+7.0 (−8.6, +23)	+0.4 (−14.4, +15.2)	−6.7 (−27.9, +14.5)	
IL-6	pg	+14.4 (+6.0, +22.9)	+1.3 (−0.2, +2.7)	−13.2 (−23.4, −3.0)	P
IL-7	pg	*+60 (+40, +70)*	+10 (0, +30)	−*50 (*−*70,* −*20)*	*P*
IL-10	pg	*+378 (+237, +520)*	−38 (−103, +28)	*−416 (−581,* *−251)*	*P*
IL-11	pg	+64 (+2.0, +126)	6.5 (−25.0, +12.1)	71 (−143, +2.0)	P
IL-18	pg	*+520 (+300, +740)*	−50 (−520, +430)	−570 (−1130, −10)	*P*
Insulin	µIU	−0.7 (−1.1, −0.2)	*−1.1 (−1.5,* −*0.7)*	−0.5 (−1.0, +0.1)	(P,*C*)
KC/GROα (CXCL1)	pg	*+680 (+460, +890)*	+40 (+40, +120)	*−640 (−900,* *−370)*	*P*
Lymphotactin (XCL1)	pg	*+144 (+101, +188)*	*+41 (+15, +68)*	*−103 (−154,* *−51)*	*P*
MCP-1 (CCL2)	pg	*+700 (+563, +837)*	+65 (+5.9, +124)	*−635 (−796,* *−474)*	*P*
MCP-3 (CCL7)	pg	*+550 (+396, +703)*	+78 (−45, +201)	*−472 (−635,* *−310)*	*P*
MCP-5 (CCL12)	pg	*+145 (+190, +100)*	+46 (+90, +2.8)	*−99 (−44,* *−153)*	*P*
M-CSF	pg	*+1180 (+780, +1590)*	+180 (−220, +580)	*−1000 (−1460,* *−540)*	*P*
MDC (CCL22)	pg	+29 (−44, +102)	−97 (−166, −28)	*−126 (−207,* *−45)*	(*C*)
MIP-1α (CCL3)	pg	+90 (+20, +200)	*+120 (+30, +220)*	+30 (−50, +110)	P,*C*
MIP-1β (CCL4)	pg	*+216 (+180, +253)*	+15 (−13, +44)	*−201 (−240,* *−163)*	*P*
MIP-1γ (CCL9)	pg	*+7420 (+4980, +9860)*	*+7230 (+4540, +10,010)*	−180 (−4100, +3730)	*P,C*
MIP-2 (CXL1)	pg	*+11.3 (+5.7, +16.9)*	+2.3 (+0.7, +5.3)	+9.0 (+15.8, +2.2)	*P*,C
MIP-3β (CCL19)	pg	*+210 (+120, +310)*	+80 (−40, +190)	−140 (−240, −40)	*P*
MMP-9	ng	*+37 (+14, +60)*	+12 (−1.3, +25)	−25 (−54, +4.1)	*P*
Myeloperoxidase	ng	*+93 (+62, +123)*	*+118 (+102, +135)*	+25 (−14, +65)	*P,C*
Oncostatin M	pg	*+100 (+70, +150)*	−10 (−20, +10)	*−120 (−160,* *−70)*	*P*
Osteopontin	pg	*+22.9 (+8.8, +37.0)*	*+81 (+52, +109)*	*+58 (+23, +93)*	*P,C*
Serum Amyloid P	µg	*+15.4 (+10.1, +20.6)*	*+14.0 (+9.5, +18.5)*	−1.4 (−8.5, +5.8)	*P,C*
SGOT	µg	+5.6 (−4.9, +16.1)	+13.7 (+2.8, +24.6)	+8.1 (−8.2, +24.4)	C
Stem Cell Factor	pg	*+68 (+39, +97)*	−6.3 (−32, +19.5)	*−74 (−115,* *−34)*	*P*
TIMP-1	pg	*+940 (+480, +1,400)*	*+980 (+630, +1,330)*	+50 (−460, +560)	*P,C*
TNF-α	pg	+20 (0, +40)	0 (−10, 0)	−20 (−50, 0)	P
VCAM-1	ng	*+405 (+257, +552)*	*+540 (+395, +685)*	+135 (−67, +337)	*P,C*
VEGF	pg	+49 (+8.5, +90)	+6.6 (−44, +57)	−43 (−88, +2.9)	P

**^a^** Measurement unit per milliliter of plasma; IU, International Unit; **^b^** P, increase during peak; C, increase during clearance; (P), decrease during peak; (C), decrease during clearance; **^c^** Italics, *p* < 0.01 by two-tailed T-test; **^d^** Abbreviations defined in Table S1; **^e^** (CXCL10), standardized chemokine designation.

## Supplementary Materials

The following are available online at www.mdpi.com/2306-7381/3/3/19/s1, Table S1: Serum protein analytes of rodent panel.

## Abbreviations

The following abbreviations are used in this manuscript:

J-H	Jarisch-Herxheimer
IFA	indirect immunofluorescence assay
PBS	phosphate-buffered saline
qPCR	quantitative polymerase chain reaction
SCID	severe combined immunodeficient
TLR	Toll-like receptor
