# Supplementary material for: Pathogen and Host Response Dynamics in a Mouse Model of Borrelia hermsii Relapsing Fever
Source: Vet Sci. 2016 Aug 30;3(3):19. doi: 10.3390/vetsci3030019 (PMC5606581; doi:10.3390/vetsci3030019)
Supplement: Supplementary file 1 [file vetsci-03-00019-s001.pdf]

Supplementary Materials: Pathogen and Host Response Dynamics in a Mouse Model of *Borrelia hermsii* Relapsing Fever

Christopher D. Crowder, Arash Ghalyanchi Langeroudi, Azadeh Shojaee Estabragh, Eric R. G. Lewis, Renee A. Marcsisin and Alan G. Barbour

Table S1. Serum protein analytes of rodent panel.

Analyte	Abbreviation	Alternative	Protein Class
Apolipoprotein A1	Apo A1		Lipoprotein component
Beta-2 Microglobulin	β-2 microglobulin		MHC II component
Calbindin			Calcium-binding
CD40			Cell receptor
CD40 Ligand	CD40L	CD154	Receptor ligand
Clusterin			Chaperone
C-reactive Protein	CRP		Acute-phase protein
Cystatin-C			Protease inhibitor
Epidermal Growth Factor	EGF		Growth factor
Endothelin-1			Vasoconstrictor
Eotaxin		CCL11	Chemokine
Factor VII			Clotting factor
Fibroblast Growth Factor-9	FGF-9		Growth factor
Fibroblast Growth Factor-2	FGF-2		Growth factor
Fibrinogen			Acute-phase protein
Glutathione S-Transferase	GST		Enzyme
Granulocyte Chemotactic Protein-2	GCP-2	CXCL6	Chemokine
Granulocyte Macrophage-Colony Stimulating Factor	GM-CSF	CSF2	Cytokine
Growth Hormone			Hormone
Growth-Regulated Alpha Protein	KC/GROα	CXCL1	Chemokine
Haptoglobin			Acute-phase protein
Interferon-gamma	IFN-γ		Cytokine
Immunoglobulin A	IgA		Immunoglobulin
Interleukin-10	IL-10		Cytokine
Interleukin-11	IL-11		Cytokine
Interleukin-12p70	IL-12p70		Cytokine
Interleukin-17	IL-17		Cytokine
Interleukin-18	IL-18		Cytokine
Interleukin-1alpha	IL-1α		Cytokine
Interleukin-1beta	IL-1β		Cytokine
Interleukin-2	IL-2		Cytokine
Interleukin-3	IL-3		Cytokine
Interleukin-4	IL-4		Cytokine
Interleukin-5	IL-5		Cytokine
Interleukin-6	IL-6		Cytokine
Interleukin-7	IL-7		Cytokine
Insulin			Hormone

Table S1. *Cont.*

Analyte	Abbreviation	Alternative	Protein Class
Inducible Protein-10	IP-10	CXCL10	Chemokine
Leptin			Hormone
Leukemia Inhibitory Factor	LIF		Cytokine
Lymphotactin		XCL1	Chemokine
Monocyte Chemoattractant Protein-1	MCP-1	CCL2	Chemokine
Monocyte Chemoattractant Protein-3	MCP-3	CCL7	Chemokine
Monocyte Chemoattractant Protein-5	MCP-5	CCL12	Chemokine
Macrophage-Colony Stimulating Factor	M-CSF	CSF1	Cytokine
Macrophage-Derived Chemokine	MDC	CCL22	Chemokine
Macrophage Inflammatory Protein-1alpha	MIP-1α	CCL3	Chemokine
Macrophage Inflammatory Protein-1beta	MIP-1β	CCL4	Chemokine
Macrophage Inflammatory Protein-1gamma	MIP-1γ	CCL9	Chemokine
Macrophage Inflammatory Protein-2	MIP-2	CXL1	Chemokine
Macrophage Inflammatory Protein-3beta	MIP-3	CCL19	Chemokine
Matrix Metalloproteinase-9	MMP-9		Enzyme
Myeloperoxidase	MPO		Enzyme
Myoglobin			Muscle protein
Lipocalin-2	LCN2	NGAL	Transport protein
Oncostatin M	OSM		Cytokine
Osteopontin	OPN		Immune modulator
"Regulation upon Activation, Normal T-cell Expressed, Secreted"	RANTES	CCL5	Chemokine
Serum Amyloid P	SAP		Acute-phase protein
Stem Cell Factor	SCF	KITLG	Cytokine
Serum Glutamic-Oxaloacetic Transaminase	SGOT		Enzyme
Tissue Inhibitor of Metalloproteinase Type-1	TIMP-1		Protease inhibitor
Tissue Factor		Thromboplastin	Clotting factor
Tumor Necrosis Factor-alpha	TNF-a		Cytokine
Thrombopoietin	TPO	THPO	Hormone
Vascular Cell Adhesion Molecule-1	VCAM-1		Adhesion protein
Vascular Endothelial Cell Growth Factor	VEGF		Growth factor
von Willebrand Factor	vWF		Acute-phase protein
